# Threitol, a Novel Functional Sugar Alcohol Biosynthesized by Engineered *Yarrowia lipolytica*, Has the Potential as a Low-Calorie Sugar-Reducing Sweetener

**DOI:** 10.3390/foods14142539

**Published:** 2025-07-20

**Authors:** Qing Li, Shuo Xu, Tong Li, Liyun Ji, Hairong Cheng

**Affiliations:** State Key Laboratory of Microbial Metabolism, School of Life Sciences and Biotechnology, Shanghai Jiao Tong University, Shanghai 200240, China; nihilist@sjtu.edu.cn (Q.L.); xushuo@sjtu.edu.cn (S.X.); litong.t2d@sjtu.edu.cn (T.L.); jiliyun@sjtu.edu.cn (L.J.)

**Keywords:** sugar alcohol, *Yarrowia lipolytica*, toxicity, gut microbiota

## Abstract

The global obesity and metabolic syndrome epidemic have accelerated demand for reduced-sugar food, prompting the food industry to adopt functional sugar alcohols as sucrose substitutes. Threitol is a four-carbon sugar alcohol and an isomer of erythritol. However, there is a scarcity of studies reporting on the edible safety of threitol. This study assessed threitol’s toxicological and metabolic properties. Acute oral administration (10 g/kg) caused no mortality or abnormalities in mice. Repeated 28-day exposure revealed no behavioral or histopathological alterations, with negative outcomes in three genotoxicity tests. Metabolic studies in rats demonstrated that the majority of ingested threitol is excreted in the urine within 24 h. Sensory evaluation indicated threitol’s sweetness equivalence to sucrose, exceeding erythritol and allulose. Notably, 16S rRNA sequencing revealed gut microbiota modulation in threitol-fed mice, indicating potential intestinal health benefits. These integrated findings establish threitol’s preclinical safety and support its development as a novel low-calorie sweetener.

## 1. Introduction

Obesity remains a significant public health issue, with the global surge in obesity prevalence partly attributed to the excessive consumption of added sugars [1]. The World Health Organization advises that added sugars should constitute less than 10% of daily calorie intake, and ideally, this should be reduced to 5% or less for optimal health [2]. Sweeteners are broadly categorized into two groups based on their origin: artificial and natural. Artificial sweeteners encompass aspartame, acesulfame, neotame, saccharin, and others, while natural sweeteners include sorbitol, erythritol, xylitol, and other sugar alcohols. However, there has been enduring controversy over the safety and health implications of artificial sugar substitutes [3,4,5]. Potential adverse effects associated with long-term use of artificial sweeteners include an increased risk of type 2 diabetes and cardiovascular disease [6]. This has led to a preference for natural sweeteners in sugar-free products. As attention to healthy diet grows, reducing-sugar and sugar-free products are becoming increasingly popular. The global market for natural sweeteners is now worth billions of dollars and is projected to continue in the coming years.

Polyols, or sugar alcohols, are appealing natural alternatives to synthetic sweeteners due to their low caloric content, minimal impact on blood glucose levels, and ability to be metabolized without insulin [7]. These properties, along with their high temperature stability, minimal reaction in acidic or alkaline environments, and lack of participation in Maillard-type browning reactions [8], make them ideal for use in food processing. As a result, they are commonly used in beverages, confectionery, and bakery products to satisfy consumer taste preferences while reducing sugar intake. However, recent studies have shown that erythritol [9,10] and xylitol [11] may cause platelet aggregation, enhance platelet reactivity, and potentially promote thrombosis. Consequently, the food industry has become wary of using these sugar alcohol sweeteners. Therefore, the development and research of new sugar alcohol sweeteners hold significantly practical application.

Threitol (1,2,3,4-butanetetetrol) is a four-carbon sugar alcohol and a diastereoisomer of erythritol. It is naturally occurring in the fungus *Armillaria mellea* and the Alaskan beetle *Upis ceramboides* [12], where it serves as an osmoprotectant and antifreeze agent under extreme low temperatures, owing to its non-specific protective effect on organisms and biomolecules. In humans, threitol might be the final product of D-xylose metabolism, a biotransformation that takes place in the liver [13].

Threitol has a limited range of applications within chemical and pharmaceutical domains. It is primarily utilized as a precursor for the creation of chiral auxiliaries in organic chemistry [14]. Additionally, it serves as a component of oxygen-sensitive pigments that are integrated into smart plastic films for food packaging [15]. Threitol is also employed as a precursor substance in the synthesis of the anticancer drug, suxamethonium, which is clinically administered to patients with ovarian cancer and for the treatment of active secondary progressive multiple sclerosis [16,17]. Immunologically, threitol is used as a precursor to the drug tributyrate, which is used in the development of anticancer drugs, and threonine ceramide (ThrCer2), which stimulates invariant natural killer T (iNKT) cells. This stimulation subsequently leads to the production of interferon-γ and interleukin-4 [18]. Lastly, threitol is employed in the synthesis of phospholipids and other artificial amphiphilic phosphates [19].

Nevertheless, there are no existing reports on the food safety of threitol in scientific literature to date. Our laboratory has developed an efficient biosynthesis method for threitol, based on prior studies [20]. This method employs glucose to synthesize threitol in large quantities using an engineered strain of *Yarrowia lipolytica*. The yield can reach up to 130 g/L, and it has already achieved large-scale production. Hence, this study aims to investigate whether threitol can be utilized as a novel low-calorie sweetener. For the first time, this study examines the in vivo toxicity and pharmacokinetics of threitol. Upon confirming the favorable safety profile of threitol, we proceeded to evaluate its sweetness in taste for the first time. Furthermore, we explored the potential effects of oral administration of threitol on the gut microbiota of mice.

## 2. Materials and Methods

### 2.1. Threitol Preparation

The threitol samples used in this study were produced by our research group utilizing fermentation with the engineered *Yarrowia lipolytica* strain previously described [20]. The fermentation medium composition was 300 g/L glucose, 6 g/L yeast extract, 2 g/L peptone, 3 g/L ammonium citrate, and 4 g/L diamine hydrogen phosphate. The cultivation was conducted at 30 °C and 600 rpm in a 10 L fermenter. Upon completion of fermentation, yeast cells were sedimented via centrifugation at 5000 rpm for 15 min. Subsequently, the supernatant underwent rotary evaporation to remove excess water. This concentrated solution was then stored at 4 °C, leading to crystal formation. These crystals were purified by multiple washes with anhydrous ethanol to remove any remaining impurities, a process repeated 3–5 times. The final product, white transparent crystals, was obtained after successive recrystallizations, and its purity was confirmed through high-performance liquid chromatography.

### 2.2. Cell Culture

Mouse embryonic fibroblasts (MEFs) and human embryonic kidney (HEK-293) cells were purchased from the Cell Bank of the Chinese Academy of Sciences (Beijing, China) and cultured in DMEM medium containing 10% fetal bovine serum and 1% penicillin plus streptomycin in a cell culture incubator at 37 °C with 5% CO_2_. The cells were digested and passed with 0.25% trypsin and 0.02% EDTA.

### 2.3. Cell Viability Assay

Cell viability was assessed by CCK-8 methods in living cells. MEFs and HEK-293 cells were inoculated in 96-well cell culture plates at 10^3^–10^4^ cells, respectively, and 6 replicate wells were set in each group. The cells were incubated in 37 °C, 5% CO_2_ cell culture incubator for 24 h and then treated with different concentrations of threitol (500, 50, 5, and 0.5 mmol/L) for 12, 24, and 48 h. The culture medium was discarded after the treatment, replaced with fresh medium, 10 μL CCK-8 solution was added to the cell wells and blank wells, and the cells were put into 37 °C cell culture incubator and incubated for 1–4 h. The culture plate was taken out and the OD_450_ value was detected with an enzyme label.

### 2.4. Animal Ethics Statement

All animal trials were performed at the Laboratory Animal Center of Shanghai Jiao Tong University, China. Mice were housed in a specific pathogen free (SPF) facility maintained at 22.0 ± 1 °C with 30–70% humidity and a light/dark phase cycle for 12 h (lights on at 07:00 a.m.). All animal experimental procedures were approved by the Institutional Animal Care and Use Committee (IACUC) of Shanghai Jiao Tong University (No. A2024409, A2024471).

### 2.5. Single Dose Acute Toxicity

Six-week-old ICR mice were randomly allocated into two groups, each comprising 10 males and 10 females. Following a week of acclimatization feeding, the control group was administered with 0.9% saline (carrier), while the test group received 10 g/kg body weight of threitol. Both preparations were administered via gavage. Mice were monitored hourly for the first 4 h and subsequently assessed daily for a duration of 14 days. Behavioral observations encompassed general activity, irritability, response to touch, twitching, tremors, convulsions, tachycardia, piloting, stereotyped movements, drowsiness, defecation, diarrhea, and urination. The body weight, water, and food consumption of the animals were evaluated daily. On the 15th day following the treatment, the mice were euthanized by inhaling CO_2_. Blood samples were then collected by cardiac puncture for hematological and biochemical analysis. Additionally, the main organs were collected and weighed, and the liver, kidneys, and spleen were selected for histopathological examination.

### 2.6. Repeated Dose Toxicity

The optimal dosage of threitol was determined based on results from the acute toxicity test. Three groups, each comprising of six 6-week-old C57BL/6J male mice, were subjected to different treatments—250 mg/kg, 500 mg/kg, and 1000 mg/kg of threitol. A control group was also maintained, which received only the vehicle (0.9% saline solution). The treatments were administered daily through gavage over a span of 28 days. Throughout the study, the animals were monitored daily for changes in body weight, behavior (as described previously), and their consumption of water and food. Upon completion of the experiment, all mice underwent an oral glucose tolerance test (OGTT). After fasting for 12 h, the mice were given 2 g/kg of glucose. The blood glucose of the mice was measured at 0, 15, 30, 60, and 120 min using a portable blood glucose meter (Roche, Mannheim, Germany). On the 29th day, urine samples were collected for analysis. Subsequently, on the 30th day, the mice were euthanized by inhaling CO_2_, and blood samples were collected by heart puncture for biochemical examination. Additionally, organs such as the liver, kidney, and spleen were harvested for both macroscopic and histological evaluations.

### 2.7. Biochemical and Hematological Analysis

The blood samples collected in EDTA-lined tubes were examined using a SYSMEX automated hematology analyzer (XN-1000V [B1]; Sysmex Corporation, Kobe, Japan). The analysis evaluated several hematological parameters, including erythrocytes, hemoglobin, hematocrit, mean corpuscular volume (MCV), and mean corpuscular hemoglobin concentration (MCHC), in addition to total and differentiated leukocyte analysis. Any remaining blood samples were collected in sterilized centrifuge tubes. These tubes were left to stand for 1–2 h at room temperature before being centrifuged at 3500 rpm for 15 min at 4 °C to separate the serum. Subsequently, biochemical parameters such as total protein, albumin, alanine aminotransferase (ALT), aspartate transaminase (AST), alkaline phosphatase (ALP), urea, and creatinine were measured using a fully automated biochemical analyzer (cobas c 311; Roche, Mannheim, Germany).

### 2.8. Histopathological Analysis

Liver, kidney, and spleen of mice were collected for histopathological analysis. The organs were fixed with 10% neutral formalin solution for 48 h, followed by dehydration through successive ethanol concentrations (70%, 80%, 95%, and 100%). Subsequently, the organs were embedded in paraffin, sectioned, and then stained with hematoxylin and eosin (H&E). The stained slices were then examined microscopically using a light microscope (ECLIPSE Ci-L; Nikon Corporation, Tokyo, Japan).

### 2.9. Genotoxicity

#### 2.9.1. Bacterial Reverse Mutation (Ames) Test

The bacterial reverse mutation test was used to evaluate the mutagenicity of threitol. The Ames test was conducted following the recommendations of OECD test guideline 471 [21] by using five bacterial strains (*Salmonella typhimurium* TA1537, TA98, TA100, TA102, and TA1535) (Molecular Toxicology, Inc., Boone, NC, USA). The highest dose of this test was set at 5000 μg/plate, and seven dose groups were set up at 3-fold dose spacing downward. Negative and positive control groups were also set up, and each group was prepared in triplicate. The negative control group used sterile water for injection. The positive control group adopted the corresponding positive control substances (including 2-aminofluorene, 4-nitroquinoline-N-oxide, cyclophosphamide, 2-aminoanthracene, diketopine, methyl methylate, sodium azide, and acridine mutagenic agent ICR191) according to different strains and experimental conditions. Hepatic microsomal enzymes (S9) induced by the combination of β-naphthoflavone and phenobarbital in Sprague-Dawley (SD) rats were used as an in vitro metabolic activation system, and were tested by the plate doping method under ±S9 test conditions.

#### 2.9.2. In Vivo Mammalian Erythrocyte Micronucleus Test

The mammalian erythrocyte micronucleus test was performed according to OECD Guideline No. 474 test [22]. Fifty ICR mice aged 6–8 weeks were randomly divided into 5 groups of 10 mice per group, half male and half female. The maximum test dose of threitol was set at 2000 mg/kg, and dose groups of 667 mg/kg and 222 mg/kg were set downward at 3-fold dose intervals. A negative control group (0.9% saline) and a positive control group (75 mg/kg cyclophosphamide) were also set up. The negative control group and threitol dose group were given orally once on the 1st day and 2nd day, respectively, and the positive control group was given intraperitoneally once on the 2nd day. The state and behavior of mice were observed during the test period. On the 3rd day, all mice were euthanized by inhaling CO_2_, and the bone marrow of the two femurs of the mice was collected to prepare bone marrow smears. The fixed bone marrow smears were stained and examined under an oil microscope. At least 500 erythrocytes were counted per animal and the ratio of polycromatic erythrocytes (PCE) to the sum of polycromatic erythrocytes and normochromatic erythrocytes (NCE) was calculated (i.e., PCE/(PCE + NCE)). Continue counting PCE up to 4000 or more, and record the number of PCE in which micronucleus appear.

#### 2.9.3. In Vitro Mammalian Chromosome Aberration Test

An in vitro mammalian chromosome aberration test was performed according to OECD Guideline No. 473 test [23]. The maximum dose in this test was set at 500 µg/mL, and two other dose groups (166.7 µg/mL and 55.6 µg/mL) were set downward at 3-fold dose intervals. There was also phosphate-buffered solution (PBS) as the negative control, and cyclophosphamide (40 µg/mL) and mitomycin C (0.5 µg/mL) as the positive control. Hepatic microsomal enzymes (S9) induced by the combination of β-naphthoflavone and phenobarbital in Sprague-Dawley (SD) rats were used as an in vitro metabolic activation system. Chinese hamster lung fibroblasts (CHL) were selected to be cultured for 24 h, and the medium in the culture flasks was discarded. For the group with S9, the treatment time was 4 ± 1 h. In the group without S9, the treatment time was 4 ± 1 h and 24 ± 2 h. For the 4 h treatment group, contact with the threitol/control preparation for 4 ± 1 h was replaced with complete medium to continue incubation for 24 h. A final concentration of 0.1 μg/mL demecolcine solution was added 4 h before harvesting the cells. Chromosomes were observed after hypotonicity, fixation, preparation and Giemsa staining. A total of 300 mid-phase cells were analyzed in each dose group and negative control group, and 100 mid-phase cells were analyzed in the positive control group. The rate of chromosomal aberrant cells was calculated for each dose group, negative control group, and positive control group.

### 2.10. In Vivo Metabolism Assay

Three healthy Wistar rats, each weighing 200–250 g, were selected for in vivo pharmacokinetic study and were in fasted for 12 h before the test. Rats were administered with 2000 mg/kg of threitol (solvent 0.9% saline) by gavage, and blood samples (0.5 mL) were collected from the retro-orbital plexus at pre-established intervals (0, 0.25, 0.5, 1, 2, 4, 8, 12, and 24 h). Each time blood was collected, rats were anesthetized by inhaling 3% isoflurane for 3–5 min in advance. The collected blood samples were transferred into heparinized microtubes. After centrifugation at 4000 rpm for 15 min at 4 °C, the supernatant was separated. Additionally, urine and fecal samples were also collected from rats every 12 h. The urine was centrifuged at 12,000 rpm for 10 min to obtain the supernatant. The feces were mixed with water and then ground with a grinder at 60 Hz for 2 min. After the experiment, the rats were euthanized by CO_2_ inhalation.

Then the pre-treated serum, urine, and feces were silanized and the concentrations of threitol in the samples were determined by GC-MS. The instrument used was ThermoFisher TRACE 1300 Gas Chromatograph (Agilent, Palo Alto, CA, USA) equipped with an MSD ISQ7000 column. The column was a DB-5MS (30 m × 0.25 mm × 0.25 μm) fused-silica capillary column. The column temperature was initially held at 80 °C for 1 min, and then it was raised to 180 °C at a rate of 20 °C/min, to 240 °C at a rate of 5 °C/min, and to 290 °C with 25 °C/min again, and finally held at 290 °C for 10 min. The injector temperature was 290 °C. The carrier gas was helium, and the column flow was 1.0 mL/min. The injection volume was 1 μL and the split ratio was 1:5. The ion source and the transfer line temperatures were 230 °C and 280 °C, respectively. The electron impact ionization mode was operated at 70 eV. The MS was operated in scan (33–500 m/z) mode. The solvent delayed at 2.5 min.

### 2.11. Gut Microbiome Analysis

Following the repeated dose toxicity experiments, fecal samples were collected from both experimental and control groups. DNA was subsequently extracted and the 16S rRNA genes were PCR amplified. These amplified DNA fragments were then used for library preparation, which were sequenced using a high-throughput sequencing platform (Illumina MiSeq/NovaSeq). Following the sequencing process, the obtained sequence data were filtered and spliced for quality control. Low quality and chimeric sequences were removed to generate optimized sequences. These optimized sequences served as the basis for OTU (Operational Taxonomic Unit) clustering analysis and species taxonomic annotation. The results of the OTU analysis were then used to perform α-diversity and β-diversity analyses. The student t-test was employed to compare the diversity index between groups. Additionally, Metastats difference analysis was utilized to identify species abundance disparities among different groups, and to pinpoint species exhibiting significant differences among groups.

### 2.12. Sensory Evaluation for Sweetness

The sweetness of a given sugar alcohol is quantified in relation to standard sucrose. In this study, we engaged five well-trained assessors to assess the sweetness of threitol. The assessment process was as follows: initial evaluation of 10% sucrose for sweetness—subsequent re-evaluation of 5% sucrose post-pasteurization—final evaluation of 5% sucrose pre-pasteurization. Comparison of sweetness intensity, speed of sweetening, and aftertaste with sucrose was scored on a scale of 1–5, with 3 being the standard for sucrose, with higher scores indicating a more intense degree of intensity. Overall proximity to sucrose was scored on a scale of 1–10. The entire evaluation spanned three rounds, conducted over a span of 10 days.

### 2.13. Statistical Analysis

Data in this study are expressed as mean ± standard deviation (SD). Statistical differences were analyzed using GraphPad Prism 10.0 applying a one-way analysis of variance (ANOVA) or unpaired Student’s *t*-test. Statistical significance is expressed as * *p* < 0.05, ** *p* < 0.01, *** *p* < 0.001, ns: not significant.

## 3. Results

### 3.1. Threitol Preparation and Characterization

**Figure 1:**
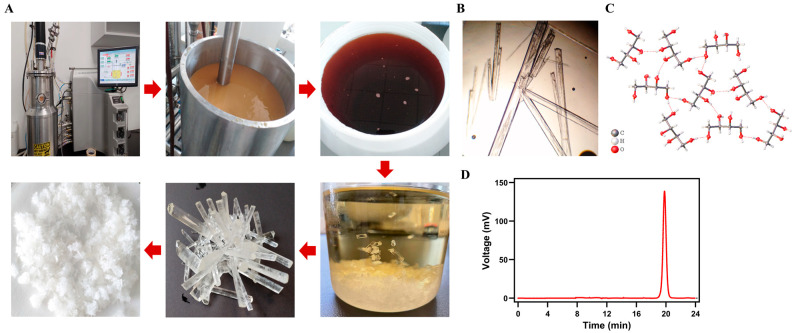
Production and identification of threitol: (**A**) Production flowchart (including fermentation, centrifugation, crystallization, decolorization, and recrystallization steps). (**B**) Crystals photographed under the microscope. (**C**) Structural identification. (**D**) Purity analysis via HPLC.

Based on the previous research of our group [20], we transitioned the preparation of threitol from shake flask to tank fermentation. The complete product preparation is depicted in Figure 1A. Samples were withdrawn every 12 h to monitor the glucose content, ending the fermentation once glucose was fully consumed. Then, the yeast cells were separated via membrane filtration, yielding clear fermentation broth. This fermentation broth was concentrated through evaporation until the soluble solids content reached 600 g/L, at which point it was cooled to 4 °C, and left to gradually form crystals, which were gradually washed with pre-cooled ultrapure water, and then re-dissolved using ultrapure water for secondary crystallization, and ultimately resulted in white pure threitol crystals. Microscopic observation revealed the crystal shape of the threitol, as shown in Figure 1B. X-ray diffraction confirmed the identification of these crystals as threitol (Figure 1C and Table S1). Concurrently, we determined the purity of the threitol samples using high-performance liquid chromatography (HPLC), finding it to be over 99.9% (Figure 1D). This indicates the sample is suitable for subsequent cell and animal testing.

### 3.2. Effect of Threitol on Cell Viability

**Figure 2:**
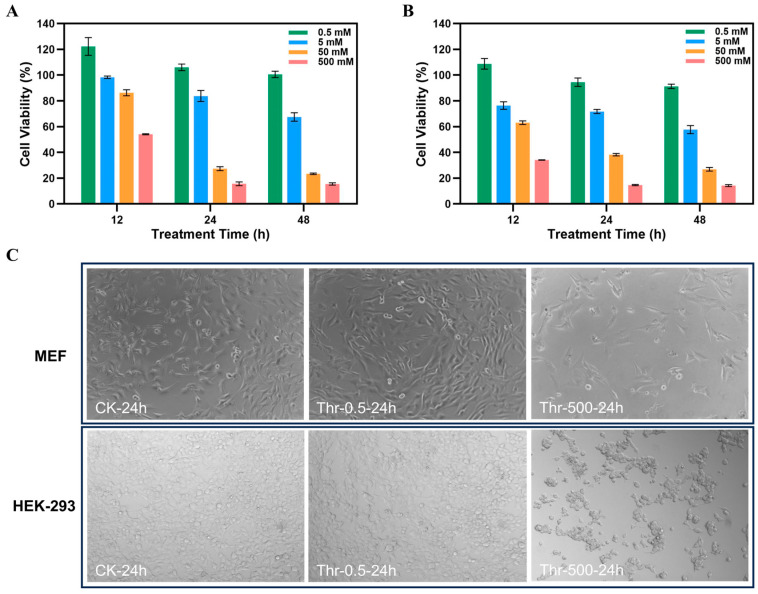
Cell viability at different concentrations (0.5, 5, 50, 500 mM) of threitol for different times (12, 24, 48 h): (**A**) MEFs. (**B**) HEK-293 cells. (**C**) Microscopic photographs of MEFs and HEK-293 cells after treatment with 0.5 mM and 500 mM threitol for 24 h.

The impact of threitol on the toxicity of mouse embryonic fibroblasts (MEFs) was investigated using the CCK-8 method. We analyzed the effect of varying threitol concentrations (0.5, 5, 50, and 500 mM) on MEF cell viability over different treatment durations (12, 24, and 48 h). Cell viability for MEFs treated with 0.5 mM threitol was recorded at 122% after 12 h, 106% after 24 h, and 101% after 48 h (Figure 2A). A decreasing trend in cell survival rate at each concentration was observed as the treatment time extended. Within the same treatment period, lower survival rates were associated with higher threitol concentrations (*p* < 0.001). These results suggest that low concentrations of threitol do not significantly affect MEF cell viability (*p* > 0.05), and indicate a dose-dependent relationship between cell survival rate and threitol concentration. Additionally, an examination of the toxic effects of varied threitol concentrations on human embryonic kidney (HEK-293) cells showed a survival rate of 109% after 12 h of treatment with 0.5 mM threitol (Figure 2B), further supporting the conclusion that low threitol concentrations do not significantly affect cell viability (*p* > 0.05). Additionally, we analyzed the quantity and morphology of MEFs and HEK-293 cells post-treatment with varying concentrations of threitol utilizing a microscope (Figure 2C). Our observations revealed that the lower concentration (0.5 mM) of threitol did not significantly impact the cells compared to the control group. However, there was a substantial reduction in cell count in the high-concentration group (500 mM), despite the cell morphology remaining unaffected.

### 3.3. Single Toxicity Test

**Figure 3:**
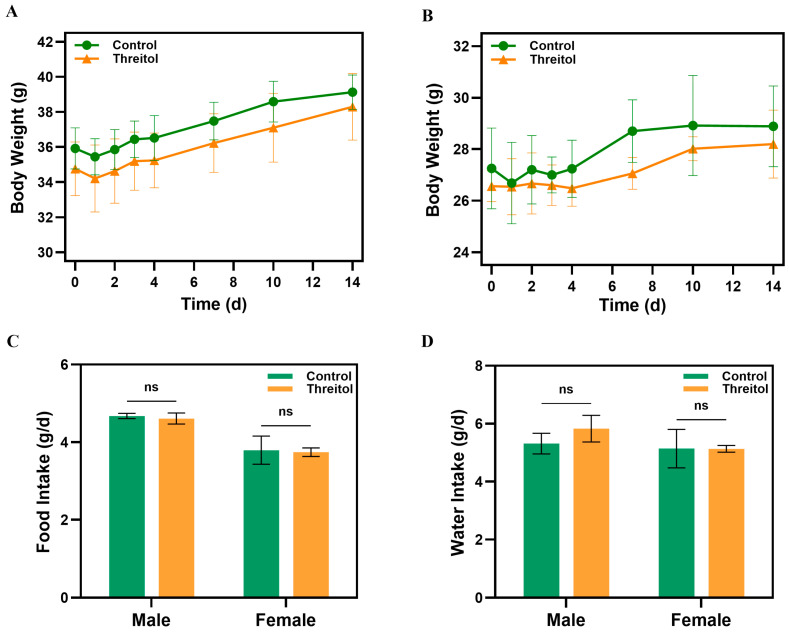
Body weight gain, food and water consumption in control and single-dose threitol (10 g/kg): (**A**) Body weight gain in male mice. (**B**) Body weight gain in female mice. (**C**) Average daily food intake. (**D**) Average daily water intake. Values are expressed as mean ± standard deviation. No significant difference (*p* > 0.05) was found in relation to the negative control.

Over the 14-day observation period, male mice exhibited an increase in body weight from 34.8 ± 1.5 g to 38.3 ± 1.9 g after a single administration of threitol (10 g/kg). Similarly, female mice demonstrated a weight gain from 26.6 ± 0.6 g to 28.2 ± 1.3 g. In the control group, the weight of male mice rose from 35.9 ± 1.2 g to 39.1 ± 1.0 g, while females increased from 27.3 ± 1.6 g to 28.9 ± 1.6 g. These results indicate that both male and female mice experienced a similar weight gain comparable to the control group, following the oral administration of 10 g/kg threitol (Figure 3A,B). Furthermore, there were no significant differences in the daily food and water intake between the threitol-treated group and the control group for either gender (Figure 3C,D). Throughout the observational duration, no abnormal behaviors or fatalities were observed in the mice. Moreover, threitol (10 g/kg) did not significantly alter the weight of any organs in the mice (Table S2) and did not manifest macroscopic changes in the brain, heart, lungs, liver, kidneys, spleen, stomach, testicles, or ovaries. Histopathologic examination of the liver, kidney, and spleen of mice in the threitol group also showed normal (Figure S1). These findings suggest that a single oral dose of 10 g/kg of threitol neither induces behavioral alterations nor signs of toxicity in experimental animals.

When compared to the control group, there was no statistical significance in the total erythrocyte counts (9.36 × 10^12^/L vs. 9.31 × 10^12^/L, 9.98 × 10^12^/L vs. 9.86 × 10^12^/L), hemoglobin content (145 g/L vs. 145 g/L, 157 g/L vs. 154 g/L), total leukocyte counts (5.28 × 10^9^/L vs. 5.46 × 10^9^/L, 5.24 × 10^9^/L vs. 5.10 × 10^9^/L), or lymphocyte proportions (71.14% vs. 71.29%, 80.99% vs. 82.59%) of male and female mice administered a single dose of threitol (10 g/kg) (Table S3). Furthermore, no significant changes were observed in the indices related to liver and kidney functions in both male and female mice in the threitol group (Table S4). These results suggest that threitol, at a dose of 10 g/kg, has negligible or no toxic effect.

### 3.4. Repeated-Dose Toxicity Assay

**Figure 4:**
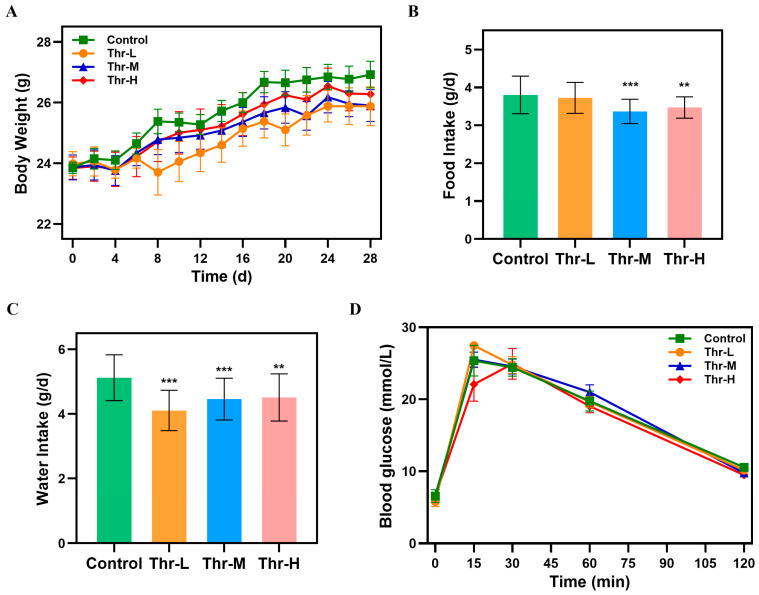
Body weight, food and water consumption, and oral glucose tolerance test (OGTT) in the control and threitol-treated groups (250, 500, and 1000 mg/kg): (**A**) Body weight gain in each group. (**B**) Average daily food intake. (**C**) Average daily water intake. (**D**) Oral glucose tolerance curve. ** *p* < 0.01, *** *p* < 0.001 vs. the control group.

The body weight growth curves for mice administered with varying doses (250, 500, or 1000 mg/kg) of threitol over a 28-day period exhibited similarities to those of the control group, with no significant deviations observed (Figure 4A). Nevertheless, it was noted that the mice in the threitol group gained 7.92%, 8.55%, and 10.23% of their body weight over four weeks, in contrast to the 12.89% weight gain observed in the control group. This suggests a potential role for threitol in modulating body weight gain, a hypothesis that needs further exploration in subsequent studies. Furthermore, the food intake for the medium- and high-dose threitol groups was recorded at 3.37 g/d and 3.47 g/d, respectively, significantly lower than the 3.80 g/d for the control group (*p* < 0.001 and *p* < 0.01). Similarly, the average daily water intake for the threitol groups showed a significant reduction (*p* < 0.01) when compared to the control group (Figure 4B,C). Furthermore, the oral glucose tolerance curves (Figure 4D) revealed no significant alterations in blood glucose fluctuations across the threitol dose groups relative to the control group (*p* > 0.05). This suggests that protracted oral administration of threitol does not induce elevated blood glucose levels.

After 28 days of treatment with threitol (at doses of 250, 500, or 1000 mg/kg), no significant changes were observed in the macroscopic features or the weights of various organs (including the brain, liver, kidney, heart, spleen, stomach, adrenal glands, thymus, and testicle) in mice when compared to the control group (Table S5). The findings suggest that repeated administration of threitol for a duration of 28 days at the concentrations evaluated (250–1000 mg/kg), did not result in behavioral alterations or any clinical signs of toxicity.

Treatment with threitol (250–1000 mg/kg) over a 28-day period did not significantly alter the blood biochemical parameters of the tested animals (Table S6). The total cholesterol level in the medium-dose threitol group was notably lower, at 2.48 ± 0.21 mmol/L, compared to the control group’s 3.05 ± 0.01 mmol/L (*p* < 0.05). Furthermore, triglyceride levels in the threitol-treated groups, administered at three different doses, were recorded at 1.30 ± 0.09 mmol/L, 1.31 ± 0.08 mmol/L, and 1.30 ± 0.07 mmol/L, respectively. These values were significantly lower than those observed in the control group, which averaged 1.77 ± 0.21 mmol/L (*p* < 0.01). Additionally, urine analysis revealed that repeated dosing of threitol throughout the 28-day treatment did not influence the ingredients detected in mouse urine compared to the control group (Table S7).

In the liver, the structure of the hepatic lobules of mice in the threitol-treated group was intact, and the hepatocytes were arranged in a radial pattern around the central vein, which did not differ from that of the control group. When compared to the control group, which received 0.9% saline treatment, the kidneys of mice in the threitol-treated group displayed no morphological alterations. They presented well-preserved curved renal tubules and glomeruli, coupled with normal-diameter Bowman’s capsule spaces. Similarly, spleens from mice treated with all concentrations of threitol demonstrated clearly delineated lymph nodes, absence of overactivation in the organ pulp, and well-defined profiles that aligned with normal conditions. These findings suggest that a 28-day treatment with threitol (250–1000 mg/kg) does not induce histopathological alterations in the liver, kidney, and spleen of mice (Figure S2).

### 3.5. In Vivo Pharmacokinetic Study

**Figure 5:**
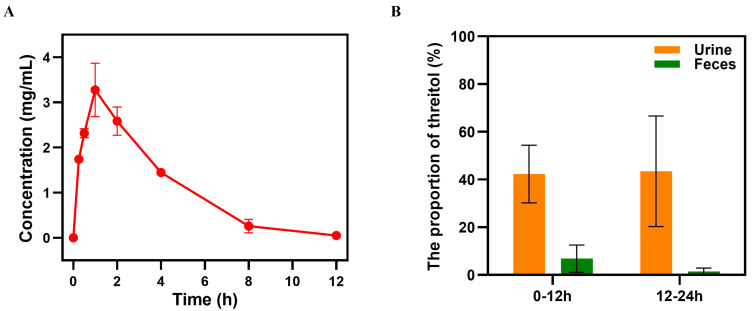
Blood, urine, and fecal levels of threitol in rats after oral administration of a single dose (2 g/kg) of threitol: (**A**) Time-dependent blood concentration of threitol. (**B**) The proportion of threitol in urine and feces collected at 12-h intervals.

To elucidate the metabolic pathway of threitol in rats, we conducted in vivo metabolism experiments using Wistar rats as a model. Following the oral administration of threitol at a dose of 2 g/kg, the plasma concentration of threitol peaked at 3.28 mg/mL within 1 h in rats. This level subsequently diminished rapidly, approaching zero by 12 h (Figure 5A). The analysis of threitol in urine and feces of rats showed that most of the threitol was excreted within 24 h after oral administration (~80% in urine and ~20% in feces) (Figure 5B). This observation indicated that the metabolic pattern of threitol is similar to that of erythritol, which is not metabolized in the body and is mainly excreted through urine without any extra metabolic load to the body.

### 3.6. Bacterial Reverse Mutation Test

In both metabolic and non-metabolic activation conditions, no precipitation was observed across all dose groups of each strain, with all background colonies appearing normal. The number of revertant mutant colonies in the positive control group significantly increased, more than doubling the count in the negative control group, thereby affirming the reliability of this test’s results. No positive mutagenic response to threitol was noted in any of the strains tested (Figure S3). Furthermore, the number of revertant mutant colonies in each dose group of every strain did not surpass twice the average number present in the negative control group in both test results, nor was there a dose-dependent incremental relationship. Thus, the bacterial revertant mutation test for threitol yielded negative results, indicating that threitol lacks mutagenicity.

### 3.7. Mammalian Erythrocyte Micronucleus Test

The PCE/RBC ratio in each dose group was not less than 20% of the negative control group, indicating that threitol had no cytotoxic effect on mouse bone marrow (Table S8). The micronucleus rate in the positive control group (male: 1.86%; female: 1.95%) was statistically and significantly higher (*p* < 0.05) than that in the negative control group (male: 0.01%; female: 0.02%). The rates of micronuclei in the low-, medium-, and high-dose groups for male mice were 0.01%, 0.02%, and 0.04% respectively, while for female mice, they were 0.02%, 0.04%, and 0.03% respectively. Compared to the negative control group, there was no significant increase in the micronucleus rate of any dose group (*p* > 0.05), and no dose-dependent correlation was observed within the micronucleus rates of the different dosed groups (*p* > 0.05). Therefore, the mammalian erythrocyte micronucleus test was negative under these experimental conditions. Threitol does not damage mammalian erythrocyte chromosomes or the mitotic apparatus, and does not induce erythrocyte micronucleus formation.

### 3.8. Mammalian Chromosomal Aberration Test

The frequency of chromosomal structural aberrations in the positive control group ranged from 25.0% to 49.0% under conditions both with and without metabolic activation systems, as shown in Table S9. This was a statistically significant increase compared to the negative control group (*p* < 0.05). In the +S9, 4 h series, the cellular rate of chromosomal structural aberrations for each dose group spanned from 0.0% to 0.33%. For the -S9, 4 h series, this rate ranged from 0.33% to 1%, and in the -S9, 24 h series, it varied from 0 to 1%. Notably, there was no statistically significant increase in the rate of chromosomal aberration cells in any threitol dose groups when compared to the negative control group (*p* > 0.05). Moreover, no quantitative chromosomal aberrations were observed across all threitol dose groups. These results suggest that the in vitro mammalian chromosome aberration test for threitol yielded negative results. Under the conditions of this test, threitol did not induce structural chromosomal aberrations in CHL cells, regardless of the presence or absence of exogenous metabolic activation systems.

### 3.9. Sweetness Evaluation

Currently, the evaluation of sweet substances primarily relies on sensory assessment [24]. The sweetness of these substances, often expressed as relative sweetness, using sucrose as a standard comparator. To determine the sweetness of various sweeteners in relation to sucrose, they are compared to an aqueous solution of sucrose of equivalent sweetness concentration [25]. Evaluation results indicate that threitol, when observed in its physical form and properties, presents as white needle-like crystals. Once dissolved in water, the solution remains colorless. In terms of its sweet characteristics, threitol exhibits a sweetening rate and aftertaste comparable to sucrose. Its overall sweetness ranges between 80 and 90% of that of sucrose. This suggests potential applications for threitol in beverage formulations to further assess its flavor profile.

### 3.10. Effects of Threitol on the Gut Microbiota in Mice

**Figure 6:**
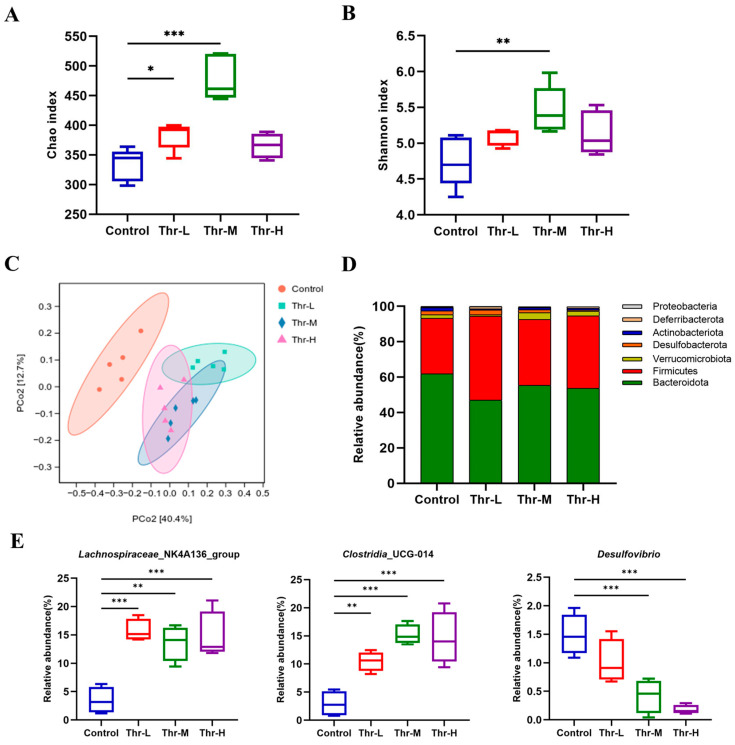
The structures and compositions of microbial communities in different groups: (**A**) Chao index. (**B**) Shannon index. (**C**) PCoA analysis chart. (**D**) The composition of gut microbiota at the phylum level. (**E**) The relative abundances of differential microbiota at the genus level. * *p* < 0.05, ** *p* < 0.01, *** *p* < 0.001 vs. the control group.

The impact of threitol on the composition and structure of the mice gut microbial community was assessed using 16S rRNA gene sequencing. The Chao index analysis revealed (Figure 6A) a significant increase in the abundance of gut microbiota in mice orally administered with threitol compared to the control group, with the Thr-M group (500 mg/kg) showing the highest increase in abundance (*p* < 0.001). The Shannon index analysis indicated (Figure 6B) a significant rise in the diversity of the intestinal microbiota in mice receiving oral threitol administration, with the Thr-M group (500 mg/kg) demonstrating the greatest increase in diversity (*p* < 0.01). To further explore the variability among different samples, downscaling of the species-annotated data was performed. The principal coordinate analysis (PCoA) clustering results indicated that the species composition of the communities in the control group and the threitol groups was almost entirely separated (Figure 6C). However, the sample dispersion was closer among the three dose treatment groups, suggesting less variation in community structure similarity. In conclusion, these results suggest that the composition and structure of the intestinal flora in mice fed with threitol have undergone changes.

The taxonomic analysis of the gut microbiome’s species composition was conducted at varying species taxonomic levels. As illustrated in Figure 6D, at the phylum level, the predominant microbiota in each group were *Bacteroidota* and *Fimicutes*. When compared to the control group, the threitol groups exhibited an increase in the abundance of *Bacteroidota* and a decrease in *Fimicutes* abundance. At the genus level (Figure 6E), there was a significant increase in the abundance of *Lachnospiraceae*_NK4A136_group and *Clostridia*_UCG-014 in the threitol groups as compared to the control group, particularly in the Thr-L group (*p* < 0.001) and the Thr-M group (*p* < 0.001). Furthermore, the Thr-M group (*p* < 0.001) and Thr-H group (*p* < 0.001) demonstrated a significantly reduced *Desulfovibrio* abundance in comparison to the control group.

## 4. Discussion

Recently, naturally occurring rare sugars have been identified as an alternative category of sweeteners. They possess potential benefits such as enhanced palatability, absence of objectionable aftertaste, and low calorie, attributed to their minimal or reduced metabolism compared to natural sugars. Upon ingestion of these sugar alcohols, their slow and incomplete intestinal absorption often leads to indirect metabolism via fermentation and degradation by intestinal flora, resulting in the production of short-chain fatty acids [26]. This lower caloric value can assist consumers in managing energy intake and facilitating weight loss [27]. Furthermore, consumption sugar alcohol has been shown to cause minimal to no increase in blood sugar levels or insulin secretion, making them a recommended option for individuals with diabetes [28,29]. Sugar alcohols also function as prebiotics and possess anti-caries properties, akin to fiber, contributing to the normalization of intestinal function [26,27,29].

Sweeteners, widely used as food additives, are a focus of public safety concerns [30]. The Food and Drug Administration (FDA) has approved several polyols for use as food additives, including erythritol, hydrogenated starch hydrolysate, isomalt, lactitol, maltitol, mannitol, sorbitol, and xylitol [7]. FDA and EFSA have reviewed and confirmed the safety of currently used low- or zero-calorie sweeteners. The primary health concern associated with polyols is the potential for gastrointestinal distress due to overdose [31], underscoring the importance of establishing an acceptable daily intake for these substances.

While threitol, a natural product catalyzed by a dehydrogenase from erythritol in the yeast *Yarrowia lipolytica*, has not been subjected to in vivo toxicity studies, thus preliminary acute toxicity tests were conducted in this study. Male and female mice administered a single, high dose of threitol exhibited no toxicity, suggesting a lethal dose (LD_50_) exceeding 10 g/kg. Based on the Globally Harmonized System of Classification (GHS) criteria, this places it in category 5, indicating very low acute toxicity or potential non-toxicity. Furthermore, the normality of hematologic and biochemical parameters post-administration suggests that this dose (10 g/kg) does not impact the hematopoietic system or protein/lipid metabolism.

The results from 28-day repeated dose toxicity assays suggest that threitol (at concentrations of 250–1000 mg/kg) exhibits negligible or non-toxic potential. The consistent body weight and consumption patterns of food and water in mice, after 28 days of threitol exposure, provide no evidence for any detrimental impact on growth or development. Furthermore, no observable indications of liver or kidney damage were detected after 28 days of treatment with threitol at tested dosage level (250–1000 mg/kg). These findings align with the normal values for liver function indicators (such as ALT, AST, and alkaline phosphatase) and kidney function markers (such as creatinine and uric acid).

Toxicological studies have often targeted morphological and functional changes in the liver, kidney, and spleen to examine the effects of compounds on metabolism, excretion, and potential immunotoxicity [32]. However, repeated dose treatments of threitol (ranging from 250 to 1000 mg/kg) showed no such changes in these organs, a finding that is congruent with results from biochemical analysis. This suggests that the physiological functions of these organs remain intact. Moreover, there were no significant changes in the weight of other organs, including the brain, lungs, and heart, after administering threitol. These results suggest that threitol exerts either a low or no toxic effect, even at high concentrations and whether administered in single or repeated doses.

The groups treated with medium and high doses of threitol exhibited a lower average daily intake in comparison to the control group. This suggests that threitol, when used as a sugar substitute, may enhance satiety and decrease appetite, thereby reducing daily energy consumption and aiding in the control of excessive weight gain. Given that over 80% of threitol is excreted in urine, it provides minimal to no energy to the body. Several randomized controlled trials have demonstrated that substituting sugar with low-calorie sweeteners can assist in managing overweight conditions [33,34]. While the consumption of low-calorie sweeteners does not directly lead to weight loss, their use as a sugar substitute can facilitate short-term weight reduction and support long-term maintenance [35]. Consequently, replacing sucrose with threitol in weight management programs might be advantageous for individuals aiming to reduce weight and regulate weight within a structured dietary framework.

Furthermore, mice that underwent oral administration of threitol for a consecutive 28-day period demonstrated reduced cholesterol and triglyceride levels. Notably, obesity—a prevalent metabolic disorder characterized by excessive body fat—improves the likelihood of contracting other diseases and health complications such as heart disease, diabetes, and hypertension [36]. However, numerous studies have discovered that fructose encourages the de novo synthesis of hepatic triglycerides, escalates the secretion of very low-density lipoproteins, and might impede peripheral lipid clearance [37,38]. Consequently, threitol’s ability to lower cholesterol and triglyceride levels could potentially enhance lipid metabolism, suggesting its potential development as a novel sweetener with associated health benefits.

Bacterial mutagenicity tests, particularly the *Salmonella* and *E. coli* reverse mutation (Ames) tests, accurately identify approximately 90% of carcinogenic chemicals and a comparable proportion of noncarcinogenic substances [39]. Micronuclei, representing the extra-nuclear genetic material of chromosome segments during cell division, can be triggered by genotoxic agents and serve as early biomarkers of genomic damage in polychromatic erythrocytes [40]. Chromosome damage is a significant indicator of genetic damage pertinent to clinical studies. Structural chromosomal aberrations arise from unrepaired direct or indirect DNA damage, or inappropriate repair leading to chromosome breaks or rearrangements [41]. The genotoxicity test results indicated that threitol is not genotoxic. No genotoxicity was observed at the concentrations tested in the bacterial reverse mutation test (up to 5000 μg/plate), the in vivo mammalian cell micronucleus test (up to 2000 mg/kg), or the in vitro mammalian chromosome aberration test (up to 500 mg/mL).

The sweetness of threitol has been noted to be 80–90% that of sucrose. In comparison [7], the sweetness of erythritol is reported to be 60–70% that of sucrose, while lactitol’s sweetness is 30–40%. Sorbitol comes in at 50–60%, and both maltitol and allulose exhibit sweetness levels of 70–80% in relation to sucrose. Consequently, the sweetness of threitol surpasses that of most sugar alcohols currently available on the market. This attribute positions threitol as a potential new generation of sugar substitutes.

The 16S rRNA sequencing results revealed a significant alteration in the composition of the intestinal microbial community in mice continuously fed threitol for 28 days. Threitol enhanced the diversity of the gut microbiota in mice by amplifying the abundance of several beneficial bacteria, including *Lachnospiraceae*_NK4A136_group, *Clostridia*_UCG-014, and *Akkermansia*, while simultaneously reducing the prevalence of certain detrimental bacteria such as *Desulfovibrio*. Previous research indicates that the *Lachnospiraceae*_NK4A136_group facilitates the production of short-chain fatty acids (SCFA), regulates nutrient absorption and hormone production within the intestine, inhibits the proliferation of pathogenic microorganisms, and is actively involved in energy metabolism [42]. SCFAs are crucial secondary metabolites of gut microbiota known to significantly influence host physiology by modulating the immune cell response [43]. An increasing body of evidence underscores the vital role of SCFAs in weight management and insulin sensitivity [44]. Consequently, it can be postulated that threitol potentially contributes to the regulation of glycolipid metabolism via its promotion of *Lachnospiraceae*_NK4A136 growth. As per previously the published literature, *Clostridia*_UCG-014 is identified as a prominent producer of short-chain fatty acids such as acetate, propionate, and butyrate [45,46]. Furthermore, *Akkermansia* has been demonstrated to be a source of SCFA producer through microbiome metabolism [47,48].

Sugar alcohols, despite their potential laxative effects, exhibit diverse impacts on the microbiome and metabolism. Research indicates that isomalt or lactitol may act as a potential prebiotic, promoting a healthy colon environment [49,50]. The term “prebiotic” denotes the food-induced augmentation of *Bifidobacteria* and *Lactobacillus* in the human gut, given that these bacteria are indicators of a well-balanced gut microbiota [51]. Isomalt, not absorbed by the small intestine, undergoes approximately 90% fermentation in the colon by the microbiome [27]. Several studies have corroborated that isomalt, in vitro, is metabolized by numerous *Bifidobacterium* strains, generating high butyrate concentrations [50]. Similarly, lactitol undergoes fermentation in the colon, producing gas and SCFA that serve as an energy source for *Bifidobacterium* and *Lactobacillus* [7]. Conversely, erythritol is quickly absorbed in the small intestine via passive diffusion, with over 90% being excreted in urine [52]. One study examining the impacts of steviol glycosides and erythritol on the gut microbiome of humans and *Cebus apella* observed a significant increase in butyric and valeric acid levels following erythritol treatment, attributing this alteration to the 10% of erythritol that reaches the colon [53]. We plan to further investigate whether threitol, primarily excreted in urine, also stimulates the gut microbiota to produce more short-chain fatty acids.

The limitation of this study is that the safety evaluation of threitol only involved acute toxicity test, 28-day repeated dose toxicity test and genotoxicity test. However, in order to conduct a more comprehensive assessment of the food safety of threitol, it is necessary to carry out studies on its teratogenicity, chronic toxicity/carcinogenicity in vivo in the future.

## 5. Conclusions

Our findings indicated that a single oral administration of threitol at a high dose (10 g/kg) and daily feeding of threitol at evaluated concentrations (250–1000 mg/kg) over 28 days did not result in any observable toxicity or histopathological alterations in mice. Pharmacokinetic assessments in rats revealed that the majority of ingested threitol is excreted through the urine. Moreover, our study suggests that threitol exhibits non-genotoxic properties, indicating a substantial margin of safety, thereby endorsing its prospective use as a sweetener in the food industry. Furthermore, the noted slight decrease in cholesterol and triglyceride levels, along with improvements in gut microbiota structure in the repeated-dose threitol-treated group, implies potential applications in ameliorating dyslipidemia and promoting a healthy intestinal environment. The development of threitol as a safe, natural sweetener with potential health benefits appears promising. Future research should explore the teratogenicity, chronic toxicity, and physiological functions of threitol in vivo.

## Data Availability

The original contributions presented in the study are included in the article and Supplementary Materials, further inquiries can be directed to the corresponding author.

## Supplementary Materials

The following supporting information can be downloaded at: https://www.mdpi.com/article/10.3390/foods14142539/s1, Figure S1: Representative photomicrographs showing liver, kidney, and spleen of mice from control and single-dose threitol (10 g/kg) groups; Figure S2: Representative photomicrographs showing liver, kidney, and spleen of mice from control and groups treated with threitol (250, 500, and 1000 mg/kg) over 28 days; Figure S3: The number of revertant colonies per plate in each treatment group of different strains under ±S9 conditions; Table S1: Crystallographic data for D-threitol; Table S2: Organ weights of mice treated orally with a single dose (10 g/kg) of threitol over 14 days; Table S3: Hematological parameters of mice treated orally with a single dose (10 g/kg) of threitol over 14 days; Table S4: Blood biochemical parameters of mice treated orally with a single dose (10 g/kg) of threitol over 14 days; Table S5: Organ weights in mice treated with threitol (250, 500, and 1000 mg/kg) over 28 days; Table S6: Blood biochemical parameters of mice treated with threitol (250, 500, and 1000 mg/kg) over 28 days; Table S7: Urine parameters of mice treated with threitol (250, 500, and 1000 mg/kg) over 28 days; Table S8: Mammalian erythrocyte micronucleus test treated with threitol; Table S9: In vitro mammalian chromosomal aberration test treated with threitol.
