# Supplementary material for: Threitol, a Novel Functional Sugar Alcohol Biosynthesized by Engineered Yarrowia lipolytica, Has the Potential as a Low-Calorie Sugar-Reducing Sweetener
Source: Foods. 2025 Jul 20;14(14):2539. doi: 10.3390/foods14142539 (PMC12294709; doi:10.3390/foods14142539)
Supplement: Supplementary file 1 [file foods-14-02539-s001.zip › foods-3745923-supplementary.pdf]

Supporting Information

Threitol, a Novel Functional Sugar Alcohol Biosynthesized by Engineered Yarrowia lipolytica, Has the Potential as a Low-Calorie Sugar-Reducing Sweetener

Qing Li, Shuo Xu, Tong Li, Liyun Ji and Hairong Cheng *

State Key Laboratory of Microbial Metabolism, School of Life Sciences and Biotechnology, Shanghai Jiao Tong
University, Shanghai 200240, China; nihilist@sjtu.edu.cn (Q.L.); xushuo@sjtu.edu.cn (S.X.);
litong.t2d@sjtu.edu.cn (T.L.); jiliyun@sjtu.edu.cn (L.J.)

* Correspondence: chrqrq@sjtu.edu.cn

Table S1. Crystallographic data for D-threitol.

Table S2. Organ weights of mice treated orally with a single dose (10 g/kg) of threitol over 14 days.

Table S3. Hematological parameters of mice treated orally with a single dose (10 g/kg) of threitol over 14 days.

Table S4. Blood biochemical parameters of mice treated orally with a single dose (10 g/kg) of threitol over 14 days.

Table S5. Organ weights in mice treated with threitol (250, 500, and 1000 mg/kg) over 28 days.

Table S6. Blood biochemical parameters of mice treated with threitol (250, 500, and 1000 mg/kg) over 28 days.

Table S7. Urine parameters of mice treated with threitol (250, 500, and 1000 mg/kg) over 28 days.

Table S8. Mammalian erythrocyte micronucleus test treated with threitol.

Table S9. In vitro mammalian chromosomal aberration test treated with threitol.

Figure S1. Representative photomicrographs showing liver, kidney, and spleen of mice from control and single-dose threitol (10 g/kg) groups.

Figure S2. Representative photomicrographs showing liver, kidney, and spleen of mice from control and groups treated with threitol (250, 500, and 1000 mg/kg) over 28 days.

Figure S3. The number of revertant colonies per plate in each treatment group of different strains under ±S9 conditions.

Table S1. Crystallographic data for D-threitol

**Parameters**	**Data**
**Empirical formula**	$C_4H_{10}O_4$
**Formula weight**	122.12
**Temperature**	173.00 K
**Crystal system**	trigonal
**Space group**	$P3_1$
	a=17.5031(3) Å α=90°
**Unit cell dimensions**	b=17.5031(3) Å β=90°
	c=4.84570(10) Å γ=120°
**Volume**	1285.63(5) $Å^3$
**Z**	9
**Density (calculated)**	1.420 g/cm^3
**Absorption coefficient**	1.108 mm^{-1}
**F(000)**	594.0
**Crystal size**	0.2×0.2×0.2 mm^3
**Radiation**	CuKα (λ=1.54178)
**2Θ range for data collection**	5.83 to 143.924°
**Index ranges**	-21≤h≤20, -21≤k≤21, -5≤l≤5
**Reflections collected**	24348
**Independent reflections**	3345 [R_{int}=0.0571, R_{sigma}=0.0384]
**Data/restraints/parameters**	3345/1/230
**Goodness-of-fit on F^2**	1.057
**Final R indexes [I>=2σ(I)]**	R_1=0.0290, wR_2=0.0779
**Final R indexes [all data]**	R_1=0.0334, wR_2=0.0791
**Largest diff. peak/hole**	0.37/-0.28 e $Å^{-3}$
**Flack parameter**	0.06(6)

Table S2. Organ weights of mice treated orally with a single dose (10 g/kg) of threitol over 14 days

**Organs**	**Males**		**Females**	
	**Control**	**Threitol (10 g/kg)**	**Control**	**Threitol (10 g/kg)**
**Brain (g)**	0.42±0.04	0.47±0.03	0.47±0.04	0.49±0.03
**Heart (g)**	0.23±0.07	0.24±0.05	0.18±0.02	0.15±0.01
**Lung (g)**	0.22±0.04	0.22±0.05	0.21±0.02	0.19±0.02
**Liver (g)**	1.60±0.21	1.57±0.15	1.09±0.18	1.11±0.11
**Kidney (g)**	0.60±0.07	0.59±0.05	0.35±0.03	0.34±0.02
**Spleen (g)**	0.11±0.02	0.14±0.05	0.11±0.02	0.10±0.02
**Stomach (g)**	0.54±0.12	0.51±0.09	0.51±0.12	0.46±0.10
**Testicle (g)**	0.23±0.04	0.25±0.02	-	-
**Ovary (g)**	-	-	0.03±0.01	0.03±0.00

Values represented by the mean ± SD. No significant difference ($p > 0.05$) was found in relation to the negative control.

Table S3. Hematological parameters of mice treated orally with a single dose (10 g/kg) of threitol over 14 days

**Parameters**	**Males**		**Females**	
	**Control**	**Threitol (10 g/kg)**	**Control**	**Threitol (10 g/kg)**
**RBC (10^{12}/L)**	9.36±0.53	9.31±0.40	9.98±0.38	9.86±0.45
**HGB (g/L)**	145±9	145±8	157±6	154±6
**HCT (%)**	46.5±3.5	46.4±2.5	47.2±1.8	46.6±2.0
**MCV (fL)**	49.6±1.7	49.9±1.2	47.3±2.0	47.3±0.9
**MCH (pg)**	15.5±0.5	15.6±0.4	15.8±0.6	15.6±0.4
**MCHC (g/L)**	313±7	313±9	333±5	331±6
**WBC (10^9/L)**	5.28±1.18	5.46±1.79	5.24±2.44	5.10±1.71
**NEUT (%)**	24.63±11.86	24.18±7.05	14.76±3.53	13.24±2.84
**LYMPH (%)**	71.14±12.48	71.29±7.46	80.99±3.79	82.59±3.35
**MONO (%)**	1.03±0.51	1.29±0.77	0.46±0.18	0.69±0.31
**EO (%)**	3.12±0.94	2.80±1.19	2.80±1.58	2.47±1.37
**BASO (%)**	0.05±0.08	0.07±0.09	0.02±0.04	0.02±0.04

RBC: total red blood cell count; HGB: hemoglobin content; HCT: hematocrit; MCV: mean corpuscular volume; MCH: mean cell hemoglobin content; MCHC: mean hemoglobin concentration; WBC: total leukocyte count; NEUT: neutrophils; LYMPH: lymphocytes; MONO: monocytes; EO: eosinophilic granulocytes; BASO: basophilic granulocytes. Values represented by the mean ± SD. No significant difference ($p > 0.05$) was found in relation to the negative control.

Table S4. Blood biochemical parameters of mice treated orally with a single dose (10 g/kg) of threitol over 14 days

Parameters	Males		Females	
	Control	Threitol (10 g/kg)	Control	Threitol (10 g/kg)
**Albumin (g/L)**	36.6±1.5	34.8±1.8	38.4±1.2	38.5±1.7
**ALT (U/L)**	50.1±11.1	43.8±16.8	29.1±12.2	27.3±3.7
**AST (U/L)**	107.9±19.7	95.2±26.4	90.5±23.1	87.4±21.6
**ALP (U/L)**	79±13	78±18	112±14	115±9
**Total protein (g/L)**	53.2±2.9	53.4±2.4	50.2±2.8	50.0±2.2
**BUN (mmol/L)**	9.1±0.6	9.1±0.8	6.7±0.8	6.9±0.7
**Creatinine (μmol/L)**	13±2	14±4	16±5	17±3

ALT: alanine aminotransferase; AST: aspartate aminotransferase; ALP: alkaline phosphatase; BUN: blood urea nitrogen. Values represented by the mean ± SD. No significant difference ($p > 0.05$) was found in relation to the negative control.

Table S5. Organ weights in mice treated with threitol (250, 500, and 1000 mg/kg) over 28 days

Organs	Control	Threitol 250 mg/kg	Threitol 500 mg/kg	Threitol 1000 mg/kg
**Brain (g)**	0.43±0.03	0.44±0.03	0.44±0.03	0.46±0.01
**Liver (g)**	1.29±0.15	1.19±0.22	1.27±0.27	1.31±0.23
**Kidney (g)**	0.34±0.02	0.31±0.02	0.33±0.01	0.34±0.02
**Heart (g)**	0.14±0.02	0.13±0.01	0.13±0.01	0.14±0.01
**Spleen (g)**	0.06±0.01	0.06±0.01	0.06±0.01	0.06±0.01
**Stomach (g)**	0.15±0.02	0.16±0.02	0.15±0.01	0.16±0.01
**Adrenal gland (g)**	0.01±0.00	0.01±0.00	0.01±0.00	0.01±0.00
**Thymus (g)**	0.03±0.00	0.03±0.01	0.03±0.00	0.03±0.01
**Testicle (g)**	0.19±0.01	0.19±0.01	0.19±0.02	0.19±0.00

Values represented by the mean ± SD. No significant difference ($p > 0.05$) was found in relation to the negative control. g: gram.

Table S6. Blood biochemical parameters of mice treated with threitol (250, 500, and 1000 mg/kg) over 28 days

Parameters	Control	Threitol 250 mg/kg	Threitol 500 mg/kg	Threitol 1000 mg/kg
**ALT (U/L)**	39.93±5.84	34.77±4.22	37.46±5.08	38.87±9.71
**AST (U/L)**	163.00±17.56	167.25±6.15	160.33±8.56	154.55±7.85
**ALP (U/L)**	106.70±2.45	100.87±7.90	102.73±6.07	109.23±6.88
**TP (g/L)**	55.10±2.42	55.50±1.25	55.53±3.49	53.33±4.52
**ALB (g/L)**	36.70±1.47	37.17±2.05	37.13±2.40	35.93±3.11
**CREA (μmol/L)**	12.00±0.00	10.33±0.58	13.67±0.58	12.33±0.58
**UREA (mmol/L)**	12.66±3.03	9.48±4.21	10.85±2.02	9.82±4.69
**T-CHO (mmol/L)**	3.05±0.01	3.04±0.38	2.48±0.21*	2.73±0.03
**TG (mmol/L)**	1.77±0.21	1.30±0.09**	1.31±0.08**	1.30±0.07**
**HDL-C (mmol/L)**	2.00±0.28	2.20±0.40	1.81±0.37	1.91±0.25

ALT: alanine aminotransferase; AST: aspartate aminotransferase; ALP: alkaline phosphatase; TP: total protein; ALB: albumin; CREA: creatinine; T-CHO: total cholesterol; TG: triglyceride; HDL-C: High density lipoprotein cholesterol. Values represented by the mean ± SD. * $p < 0.05$, ** $p < 0.01$ vs. the control group.

Table S7. Urine parameters of mice treated with threitol (250, 500, and 1000 mg/kg) over 28 days

Parameters	Control	Threitol 250 mg/kg	Threitol 500 mg/kg	Threitol 1000 mg/kg
**URO (mg/dL)**	normal	normal	normal	normal
**BLD (mg/dL)**	-	-	-	-
**BIL (mg/dL)**	-	-	-	-
**KET (mg/dL)**	-	-	-	-
**GLU (mg/dL)**	-	-	-	-
**PRO (mg/dL)**	-	-	-	-
**NIT**	-	-	-	-
**LEU (c/uL)**	-	-	-	-
**pH**	7.3±0.3	7.2±0.4	7.1±0.2	7.2±0.3
**SG**	1.022±0.005	1.019±0.004	1.021±0.006	1.020±0.003

URO: urobilinogen; BLD: blood in urine; BIL: bilirubin; KET: ketone body; GLU: glucose; PRO: protein; NIT: nitrite; LEU: leukocyte; SG: specific gravity. “-” means that the item was not detected.

Table S8. Mammalian erythrocyte micronucleus test treated with threitol

Gender	Dosage (mg/kg)	PCE	MN-PCE	Micronucleus rate (%)	PCE/RBC (%)
**Male**	Negative control	20051	2	0.01±0.01	53.08±1.08
	222	24030	2	0.01±0.01	49.76±2.67
	667	24121	6	0.02±0.03	51.00±1.75
	2000	24061	11	0.04±0.02	50.92±2.82
	Positive control	24122	449	1.86±0.11*	48.00±3.00
**Female**	Negative control	20055	4	0.02±0.02	52.52±1.77
	222	24055	4	0.02±0.02	51.24±0.95
	667	24132	10	0.04±0.02	50.19±1.22
	2000	24048	8	0.03±0.02	49.64±4.34
	Positive control	24086	470	1.95±0.11*	48.80±3.74

PCE: polychromatic erythrocytes; NCE: normochromatic erythrocytes; red blood cell (RBC)=PCE+NCE; MN-PCE: polychromatic erythrocytes containing micronucleus. * $p < 0.05$ vs. the negative control group.

Table S9. *In vitro* mammalian chromosomal aberration test treated with threitol

**Concentration (μg/mL)**	**Number of chromosomal structural aberrations**			**Chromosome aberration rate (%)**		
	**+S9, 4 h**	**-S9, 4 h**	**−S9, 24 h**	**+S9, 4 h**	**-S9, 4 h**	**−S9, 24 h**
**Negative control**	0	0	0	0.00	0.00	0.00
**55.6**	0	1	0	0.00	0.33	0.00
**166.7**	1	2	0	0.33	0.67	0.00
**500**	1	3	3	0.33	1.00	1.00
**CP**	25	-	-	25.00*	-	-
**MMC**	-	39	49	-	39.00*	49.00*

CP: cyclophosphamide; MMC: mitomycin C. * $p < 0.05$ vs. the negative control group.

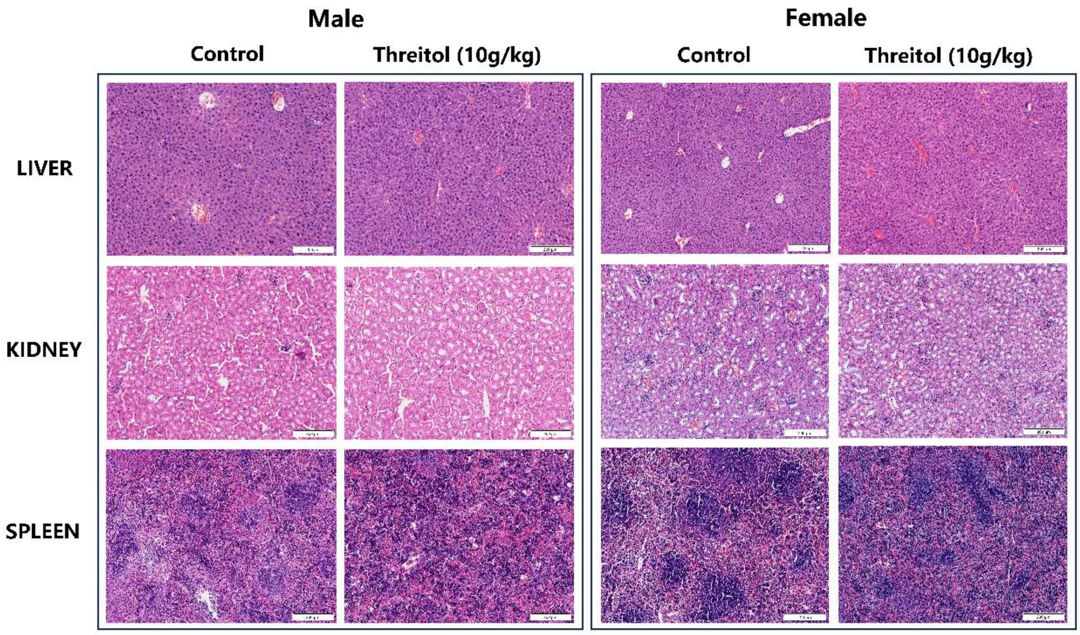

Figure S1. Representative photomicrographs showing liver, kidney, and spleen of mice from control and single-dose threitol (10 g/kg) groups.

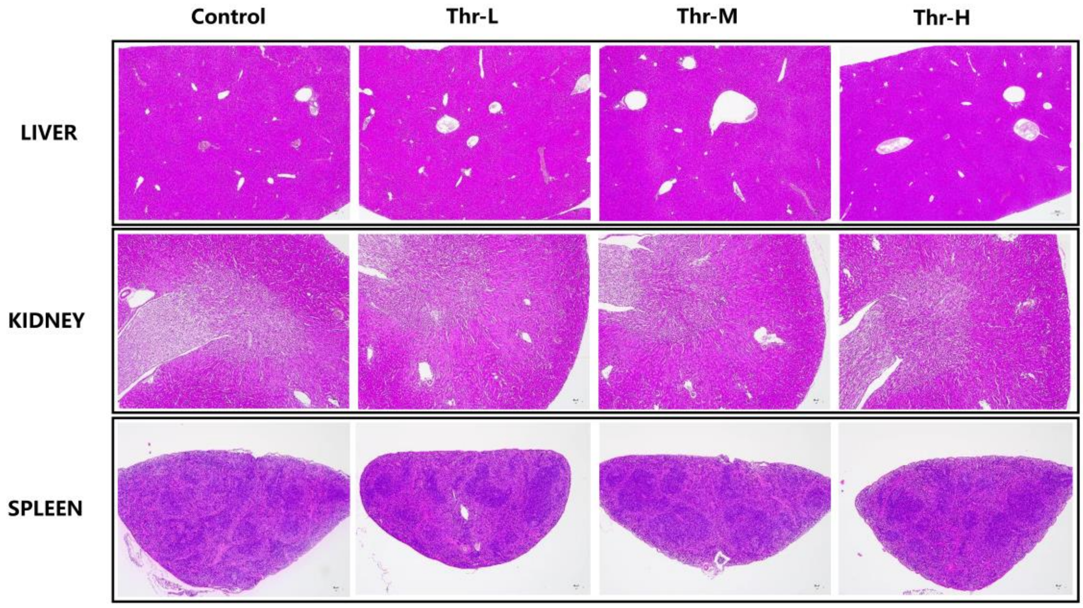

Figure S2. Representative photomicrographs showing liver, kidney, and spleen of mice from control and groups treated with threitol (250, 500, and 1000 mg/kg) over 28 days.

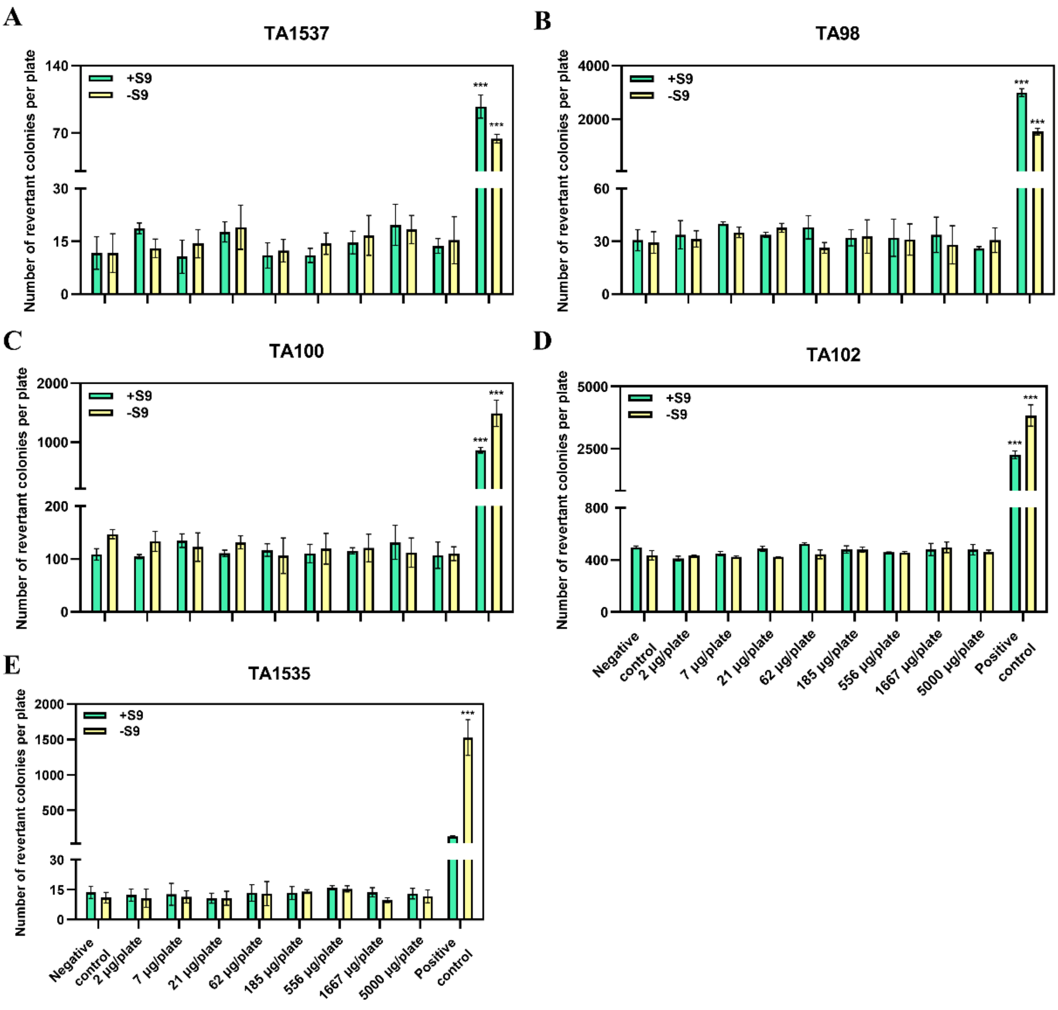

Figure S3. The number of revertant colonies per plate in each treatment group of different strains under ±S9 conditions. (A) TA1537. (B) TA98. (C) TA100. (D) TA102. (E) TA1535. All groups were set up with 3 parallel plates and the dose volume was 0.1 mL/plate. Positive control (from A to E) was: 2-aminoanthracene (3 μg/plate)/acridine mutagen ICR191 (100 μg/plate) [A]; 2-aminofluorene (20 μg/plate)/diketopine (50 μg/plate) [B]; 2-aminofluorene (20 μg/plate)/methyl methanesulfonate (1 μL/plate) [C]; 4-nitroquinoline N-oxide (20 μg/plate)/methyl methanesulfonate (1 μL/plate) [D]; cyclophosphamide (200 μg/plate)/sodium azide (2 μg/plate) [E]. Values represented by the mean ± SD. *** $p < 0.001$ vs. the negative control group.
